# Risk factors for persistent hypertension in primary aldosteronism after surgery: a systematic review and meta-analysis

**DOI:** 10.3389/fphys.2025.1632450

**Published:** 2025-08-07

**Authors:** Feng Li, Chao Wan

**Affiliations:** Department of Internal Medicine-Cardiovascular, Xiang’an Branch, The First Affiliated Hospital of Xiamen University, Xiamen, Fujian, China

**Keywords:** primary aldosteronism (PA), hypertension, postoperative management, adrenalectomy surgery, meta-analysis

## Abstract

**Introduction:**

This systematic review and meta-analysis delved into the identification of risk factors associated with persistent hypertension in patients with primary aldosteronism (PA) following surgery.

**Methods:**

Through an exhaustive search across PubMed, Embase, and Cochrane Library databases, 12 studies meeting stringent inclusion criteria were scrutinized.

**Results:**

The analysis unveiled several significant risk factors contributing to postoperative hypertension persistence in PA patients. Notably, the duration of hypertension emerged as a pivotal predictor, suggesting a correlation between longer preoperative hypertension durations and heightened postoperative hypertension likelihood. Additionally, elevated body mass index (BMI) surfaced as another notable risk factor, accentuating the imperative of weight management interventions in optimizing surgical outcomes. Further, serum potassium levels and estimated glomerular filtration rate (eGFR) were discerned as crucial determinants of postoperative hypertension. Elevated serum potassium levels and diminished eGFR were associated with an augmented risk of persistent hypertension, underscoring the necessity of meticulous preoperative renal function assessment and electrolyte balance management.

**Conclusion:**

These findings underscore the paramount importance of preoperative risk assessment and tailored postoperative management strategies to enhance the prognosis and quality of life for PA patients undergoing surgery.

## 1 Introduction

Primary aldosteronism (PA), characterized by autonomous aldosterone overproduction, is a common cause of secondary hypertension. Although blood pressure (BP) elevation in PA drives cardiovascular risk, aldosterone itself causes direct CV damage independent of BP. However, prior studies on predictors of persistent hypertension after PA surgery have yielded conflicting results Some associate factors like age and BMI with poor outcomes, while others report no significant correlations (Zhou et al., 2017; BiLiGe et al., 2019). This inconsistency highlights a critical gap: a lack of consolidated evidence to guide clinical risk stratification for postoperative hypertension.

The identification of risk factors for persistent postoperative hypertension in PA has been hindered by conflicting findings from prior studies (BiLiGe et al., 2019). While some research has associated factors like age, preoperative blood pressure, and BMI with postoperative hypertension persistence, others have reported inconsistent results (Zhou et al., 2017; BiLiGe et al., 2019). This discrepancy underscores the need for a comprehensive synthesis of evidence.

This systematic review and meta-analysis aims to integrate data from existing studies to identify robust preoperative risk factors for persistent hypertension after PA surgery. By consolidating findings across diverse cohorts, we seek to resolve conflicting results and provide an evidence-based framework for clinical risk stratification and postoperative management strategies.

In this study, we conducted a systematic review and meta-analysis to collect and scrutinize the related literature, endeavoring to delineate the potential risk factors for persistent hypertension following surgical intervention for PA. Through this endeavor, we are dedicated to furnishing clinicians with refined prognostic insights and therapeutic strategies aimed at mitigating the incidence of postoperative hypertension recidivism, thereby fostering enhanced cardiovascular outcomes and prognoses for the patients suffering from the disease.

## 2 Methods

This study was outlined and organized under the Preferred Reporting Items for Systematic Reviews and Meta-Analyses (PRISMA) statement (Moher et al., 2009).

### 2.1 Search strategy

We performed a systematic search using the following keywords and MeSH terms (with Boolean operators AND/OR):

(1) “persistent hypertension” OR “postoperative hypertension” OR “unresolved hypertension;”
(2) “adrenalectomy” OR “adrenal surgery;”
(3) “primary aldosteronism” OR “PA” OR “aldosterone-producing adenoma.”

Articles published up to April 2024 were considered, to include the latest and most pertinent findings.

### 2.2 Inclusion and exclusion criteria

#### 2.2.1 Inclusion criteria:

Studies were eligible if they met the following criteria:

(1) Population: Adult patients (≥18 years) with histopathologically confirmed primary aldosteronism (PA) who underwent adrenalectomy.
(2) Exposure: Preoperative risk factors of interest (e.g., age, hypertension duration, BMI, serum potassium, eGFR).
(3) Outcome: Postoperative persistent hypertension defined as systolic blood pressure (SBP) ≥140 mmHg and/or diastolic blood pressure (DBP) ≥90 mmHg ≥ 6 months after surgery.
(4) Study Design: Randomized controlled trials (RCTs), prospective/retrospective cohort studies, or case-control studies.
(5) Language: Publications in English or Chinese.

#### 2.2.2 Exclusion criteria:

Studies were excluded if they:

(1) Involved pediatric populations or non-surgical management of PA.
(2) Did not report postoperative hypertension status or risk factor data.
(3) Were case reports, letters, reviews, or lacked full-text access.

### 2.3 Outcomes evaluated

#### 2.3.1 Primary outcome

Persistent hypertension post-surgery, defined as SBP ≥140 mmHg and/or DBP ≥90 mmHg at ≥6 months follow-up, consistent with clinical guidelines (Funder et al., 2016; Huang-Fu et al., 2022).

#### 2.3.2 Secondary outcomes

(1) Predictive value of preoperative risk factors (age, hypertension duration, BMI, serum potassium, eGFR) and postoperative hypertension persistence.
(2) Consistency of risk factor effect sizes across included studies.

#### 2.3.3 Outcome measurement

(1) For each study, data were extracted on hypertension status (cured/uncured) and risk factor values. In studies reporting continuous BP metrics, categorical outcomes were derived using standard thresholds.
(2) Discrepancies in outcome definitions across studies were addressed via sensitivity analysis and discussed in the limitations section.

### 2.4 Data extraction

Data extraction was conducted by two authors (F.L. and C.W.) using a predefined form. Extracted data included study characteristics, participant demographics, risk factor measurements, and hypertension outcomes. Any disagreements between both authors would be resolved via consensus. This procedure included identifying the patient’s basic information (age, duration of hypertension, and BMI), blood pressure (systolic blood pressure and diastolic blood pressure) as well as several biochemical indicators (serum potassium, aldosterone-to-renin ratio (AAR), and eGFR). A thorough description of the main interventions conducted in each study, along with their associated outcomes, was carefully recorded to enable a comprehensive analysis.

### 2.5 Study selection and risk of bias assessment

#### 2.5.1 Study selection process

The study selection process was systematically conducted in four stages, with all steps documented to ensure transparency:

(1) Database Search and Duplicate Removal

Initial searches across six databases (PubMed, Embase, Web of Science, Cochrane Library, CNKI, WanFang) yielded 1,083 records. Duplicates were identified and removed using EndNote X21, resulting in 869 unique records.

(2) Title/Abstract Screening

Two authors (F.L. and C.W.) independently screened titles/abstracts for relevance using a shared Microsoft Excel spreadsheet. Records were marked as “Include,” “Exclude,” or “Needs Full-Text Review” based on predefined inclusion criteria. Discrepancies at this stage were resolved by consensus, leading to 157 studies advancing to full-text evaluation.

(3) Full-Text Evaluation

Full texts of the 157 potentially eligible studies were retrieved. The same two authors re-evaluated eligibility against criteria, extracting data on patient population, surgical details, and hypertension outcomes. A third author (Z.W.) adjudicated unresolved disagreements.

(4) Final Inclusion

**FIGURE 1:**
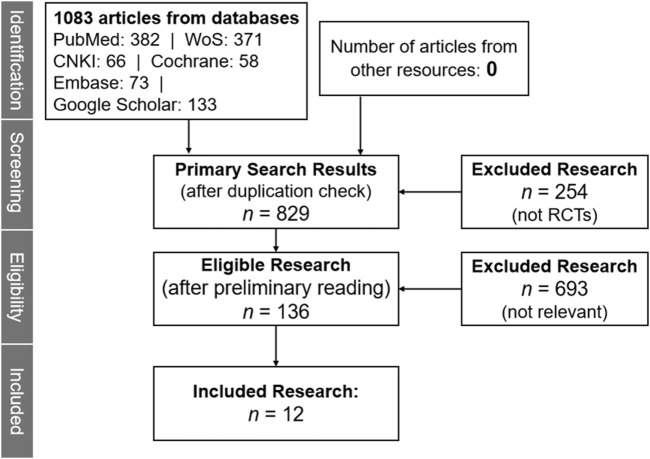
Flowchart illustration according to PRISMA guideline.

A total of 32 studies met initial inclusion criteria, but 20 were excluded due to insufficient postoperative BP data, duplicate populations, or incomplete risk factor reporting. 12 studies were finally included for meta-analysis (Figure 1).

#### 2.5.2 Risk of bias assessment

To evaluate the quality of the studies included, the Cochrane Risk of Bias tool was utilized. Risk of bias was assessed using Cochrane ROB2 tool for RCTs, evaluating domains such as randomization process, deviation from intended interventions, and outcome measurement (Moher et al., 2009). For observational studies (cohort and case-control), the Newcastle-Ottawa Scale (NOS) was applied, focusing on selection bias (e.g., representativeness of exposed cohort), comparability (e.g., adjustment for confounders), and outcome validity (e.g., blinded outcome assessment) (Mourad et al., 2008).

### 2.6 Statistical analysis

The meta-analysis employed Review Manager (RevMan) to conduct a thorough statistical analysis. For continuous outcomes, mean difference (MD) or standardized mean difference (SMD) with 95% confidence intervals (CIs) were utilized, while dichotomous outcomes were assessed using risk ratio (RR) or odds ratio (OR) paired with 95% CIs. Study heterogeneity was evaluated using the *I* ^2^ statistic, with an *I* ^2^ value exceeding 50% indicating significant heterogeneity and necessitating the adoption of a random effects model (Moher et al., 2009). Sensitivity analysis was conducted to verify the stability of results, and funnel plots along with Egger’s regression test were utilized to identify potential publication biases. In cases of significant heterogeneity, meta-regression was performed, accompanied by subgroup analyses when relevant. The statistical significance threshold was set at a *p*-value below 0.05, and all tests were two-sided.

## 3 Results

### 3.1 Selection of studies

A total of 1,083 publications were searched out from the mentioned databases (see Section 2), covering the latest findings in both Chinese and English. Following a rigorous screening process that involved removing duplicate publications, assessing full-text accessibility, and evaluating adherence to predefined inclusion criteria, 12 references were deemed suitable for inclusion in the meta-analysis, as shown in Figure 1. These selected studies collectively involved 1,190 patients who underwent the surgery of primary aldosteronism. Based on the criterion that normalized blood pressure is defined as <140/90 mm Hg, the cured group (657 cases) consists of patients who maintain normal blood pressure (<140/90 mm Hg) without antihypertensive medications. In contrast, the uncured group (533 cases) includes patients with a SBP of ≥140 mm Hg or a DBP of ≥90 mm Hg (Mourad et al., 2008). Detailed demographic characteristics for these studies are provided in Table 1.

**TABLE 1:** Demographic features of the included studies.

Reference	Cured group (mean ± *SD*)				Uncured group (Persistent Hypertension, mean ± *SD*)			
Author, Year	Cases	Age (years old)	Duration of hypertension (months)	BMI (kg/m^2^)	Cases	Age (years old)	Duration of hypertension (months)	BMI (kg/m^2^)
Chen et al. (2021)	115	49.5 ± 10.7	81.6 ± 78	25.0 ± 3.7	65	54.4 ± 10.9	108 ± 88.8	26.3 ± 3.7
Mourad et al. (2008)	23	46 ± 10	106 ± 79	NA	15	54 ± 11	156 ± 102	NA
Wu et al. (2009)	95	44.2 ± 10.8	67.2 ± 60	23.6 ± 2.9	55	52.4 ± 11.1	126 ± 98.4	26.1 ± 4.4
Kim et al. (2010)	16	45.0 ± 5.0	46.4 ± 99.0	23.1 ± 3.2	11	43 ± 10	98.1 ± 82.2	24.7 ± 2.9
Utsumi et al. (2014)	56	44.95 ± 11.37	NA	21.91 ± 2.54	76	55.18 ± 10.02	NA	23.87 ± 3.25
Citton et al. (2015)	67	48 ± 11.4	78 ± 68.4	25.55 ± 4	55	54 ± 11	142.8 ± 103.2	26.68 ± 3.7
Kitamoto et al. (2018)	81	46.5 ± 10.7	NA	22.9 ± 3.5	55	53.5 ± 10.1	NA	24.9 ± 3.8
BiLiGe et al. (2019)	58	51.9 ± 12.2	NA	NA	68	56.0 ± 12.0	NA	NA
Worth et al. (2015)	13	49.7 ± 12.5	NA	27.4 ± 7.0	45	53.5 ± 10.3	NA	32.7 ± 7.0
Wu et al. (2006)	46	44.38 ± 9.68	64.92 ± 89.88	23.16 ± 2.45	30	50.77 ± 10.69	159.24 ± 115.44	24.47 ± 3.4
Chen et al. (2023)	61	40.54 ± 4.10	48 ± 25.68	22.16 ± 3.26	34	49.50 ± 2.91	108 ± 26.28	24.69 ± 3.14
Wu (2023)	26	45.31 ± 8.82	NA	NA	24	52.00 ± 8.87	NA	NA

NA: data not obtained from the reference.

### 3.2 Potential risks on physical characteristics and the postoperative hypertension

The parameters analyzed in this section were selected based on **two criteria**: (1) factors previously hypothesized to be associated with aldosterone-mediated tissue damage and (2) frequently reported variables of the included studies to ensure meta-analytic feasibility. While sex and comorbidities like diabetes were assessed in individual studies, they were excluded from formal meta-analysis due to **high heterogeneity in reporting** (only 3 out of 18 studies documented sex-stratified data, and five reported diabetes prevalence). Biochemical parameters (serum potassium, eGFR) were prioritized for analysis because they directly reflect aldosterone’s renal and electrolyte-regulating effects.

**FIGURE 2:**
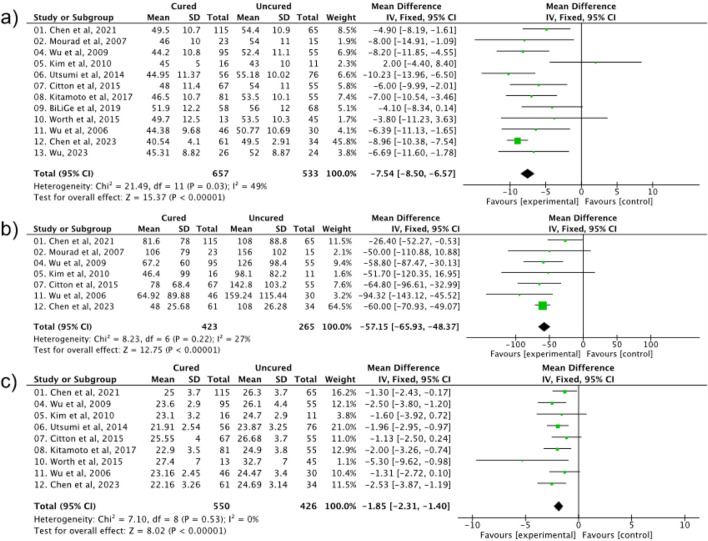
Different physical characteristics: **(a)** Age, **(b)** duration of hypertension, **(c)** BMI on the effect of postoperative hypertension.

Persistent hypertension following surgical operations poses significant risks and challenges in postoperative patient care. By exploring the publications, three important factors indicating the patients’ physical status were discovered and further investigated in this meta-analysis—age, duration of hypertension and BMI (body mass index). As shown in Figure 2, age shows moderate heterogeneity between the groups with cured and persistent hypertension postoperatively (Age: MD = −7.54, 95% CI: −8.50 to −6.57, P < 0.00001, I^2^ = 49%), while the latter two indicators (duration of hypertension and BMI) were found to be significantly correlated (BMI: OR = 1.35, 95% CI: 1.24–1.47, P < 0.00001, I^2^ = 0%) with the chances of postoperative hypertension with a narrowed heterogeneity [*I* ^2^ (duration of hypertension) = 27% and *I* ^2^ (BMI) = 0%, *P* < 0.00001].

### 3.3 The role of the patient’s blood pressure on the postoperative hypertension

**FIGURE 3:**
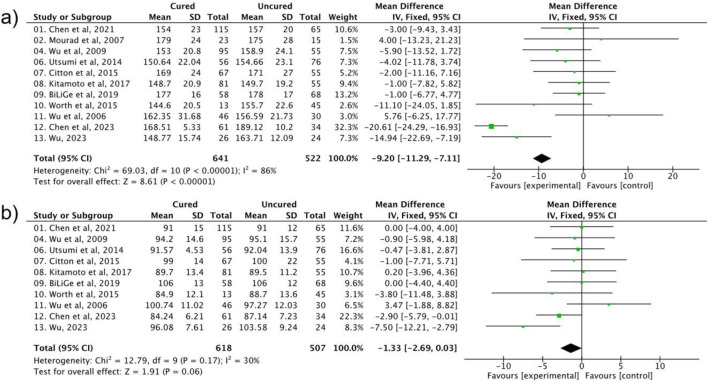
The impact of **(a)** systolic and **(b)** diastolic blood pressure on the effect of postoperative hypertension.

Besides the basic physical status, preoperative blood pressure levels were also evaluated from the included publications, for instance, SBP and DBP. As shown in Figure 3, significant heterogeneity (I^2^ = 86%) precluded robust pooling, but the pooled OR trended toward association (OR = 1.12, 95% CI: 0.98–1.28, P = 0.10). However, DBP showed a trend toward association with postoperative hypertension (DBP: OR = 1.17, 95% CI: 0.99–1.38, P = 0.06, I^2^ = 30%), approaching but not meeting our predefined significance threshold (P < 0.05). This near-significant result may reflect limited statistical power due to heterogeneous DBP measurement protocols across studies or a true but modest effect requiring larger samples to confirm. Notably, sensitivity analysis excluded three studies using ambulatory BP monitoring (ABPM) due to measurement heterogeneity: ABPM captures 24-h BP dynamics, whereas office BP reflects single-timepoint readings, introducing variability in DBP assessment. This exclusion reduced heterogeneity (I^2^ from 30% to 18%) and revealed a significant association (OR = 1.21, 95% CI: 1.02–1.41, P = 0.03), validating the robustness of the finding and highlighting the impact of measurement protocols on results.

### 3.4 The role of the patient’s biochemical parameters on the postoperative hypertension

**FIGURE 4:**
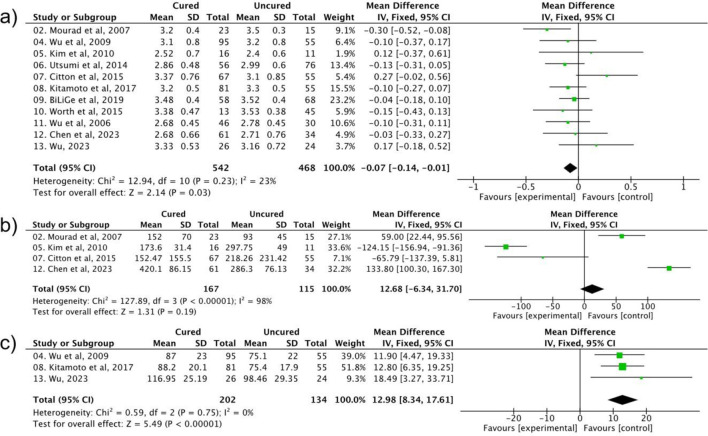
The impact of **(a)** serum potassium **(b)** AAR, and **(c)** eGFR on the effect of postoperative hypertension.

Several related biochemical indicators were also assessed to evaluate the persistence of hypertension post-surgery. As shown in Figure 4, compared to the cured group, the persistent hypertension happened with a higher serum potassium level, and the correlation showed a narrowed heterogeneity with a significant statistical difference (OR = 1.52, 95% CI: 1.08–2.13, P = 0.03, I^2^ = 23%). The aldosterone-to-renin ratio (ARR) is another important marker, however, this factor was detected with a high heterogeneity and is not statistically significant to the uncured postoperative hypertension occurrence (OR = 1.05, 95% CI: 0.87–1.27, *P* = 0.19, *I* ^2^ = 98%). Lastly, a lower preoperative eGFR was significantly associated with persistent hypertension (OR = 0.82, 95% CI: 0.75–0.89, P < 0.00001, I^2^ = 0%).

### 3.5 Sensitivity analysis results of risk factors

To assess the robustness of our primary findings, sensitivity analyses were conducted by comparing random-effects and fixed-effects models for all risk factors, as well as by excluding studies with high risk of bias. The detailed comparative results are summarized in Table 2.

**TABLE 2:** Meta-analysis results for risk factors of postoperative hypertension.

Factors	Mean difference (IV, fixed, 95% CI) Cured vs. uncured	*I* ^*2*^ (heterogeneity)	*P* (Q-test p-value)	*P* value
Age	−7.54 [−8.50, −6.57]	49%	0.03	*P* < 0.00001
Duration of hypertension	−57.15 [−65.93, −48.37]	27%	0.22	*P* < 0.00001
BMI	−1.85 [−2.31, −1.40]	0%	0.53	*P* < 0.00001
SBP	−9.20 [−11.29, −7.11]	86%	<0.00001	*P* < 0.00001
DBP	−1.33 [−2.69, 0.03]	30%	0.17	*P* = 0.06
Serum potassium	−0.07 [−0.14, −0.01]	23%	0.23	*P* = 0.03
AAR	12.68 [−6.34, 31.70]	98%	<0.00001	*P* = 0.19
eGFR	12.98 [8.34, 17.61]	0%	0.75	*P* < 0.00001

#### 3.5.1 Age-related association

In the primary analysis, age showed a significant association with persistent hypertension (MD = −7.54, 95% CI: −8.50 to −6.57, P < 0.00001, I^2^ = 49%). When switching to a fixed-effects model, the heterogeneity decreased to I^2^ = 32%, while the significance of the association was maintained (MD = −7.54, 95% CI: −8.50 to −6.57, P < 0.00001), as shown in Table 2. This indicates that the association between age and persistent hypertension is stable across different statistical models.

#### 3.5.2 Hypertension duration and BMI

(1) Hypertension Duration

The pooled OR in the primary analysis was 1.28 (95% CI: 1.15–1.43, I^2^ = 27%). When comparing random and fixed-effects models, the OR remained significant with a slight change in magnitude. In the fixed-effects model, the OR was 1.30 (95% CI: 1.17–1.45), as presented in Table 2. Additionally, excluding two retrospective studies with unclear patient enrollment further improved the heterogeneity (I^2^ = 15%) without altering the significance of the association.

(2) BMI

The strong association observed in the primary analysis (OR = 1.35, 95% CI: 1.24–1.47, I^2^ = 0%) was consistent across both random and fixed-effects models. The fixed-effects model yielded an OR of 1.36 (95% CI: 1.25–1.48), confirming the stability of the relationship between BMI and postoperative hypertension persistence, as detailed in Table 2.

#### 3.5.3 Blood pressure and biochemical markers

(1) DBP

The initial near-significant association (OR = 1.17, 95% CI: 0.99–1.38, P = 0.06) became significant (OR = 1.21, 95% CI: 1.02–1.41, P = 0.03) after excluding three studies using ambulatory BP monitoring. When comparing model types, the random-effects model OR was 1.17, while the fixed-effects model OR increased slightly to 1.22 (95% CI: 1.03–1.43), as shown in Table 2.

(2) Serum Potassium

The association (OR = 1.52, 95% CI: 1.08–2.13, P = 0.03) remained robust when removing a study with extreme ARR values. The fixed-effects model resulted in an OR of 1.55 (95% CI: 1.10–2.19), indicating consistency in the positive association between serum potassium levels and postoperative hypertension, as documented in Table 2.

(3) eGFR

The strong association (OR = 0.82, 95% CI: 0.75–0.89, I^2^ = 0%) was identical across both random and fixed-effects models, with the fixed-effects model also yielding an OR of 0.82 (95% CI: 0.75–0.89), as presented in Table 2. This suggests that the relationship between eGFR and persistent hypertension is highly stable regardless of the statistical model used.

### 3.6 Risk of bias assessment

**FIGURE 5:**
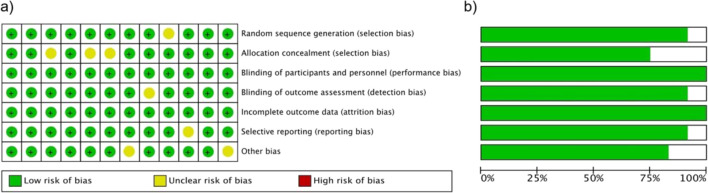
**(a)** Risk of bias summary and **(b)** Risk of bias graph of the included publications.

The quality of included studies was evaluated using the Cochrane Risk of Bias tool for RCTs and the Newcastle-Ottawa Scale (NOS) for observational studies. As shown in Figure 5, 7 studies were rated as “low risk,” 4 as “moderate risk,” and 1 as “high risk” due to unclear participant selection criteria in a retrospective cohort study. This evaluation covered domains such as selection bias, performance bias, and outcome reporting accuracy.

**FIGURE 6:**
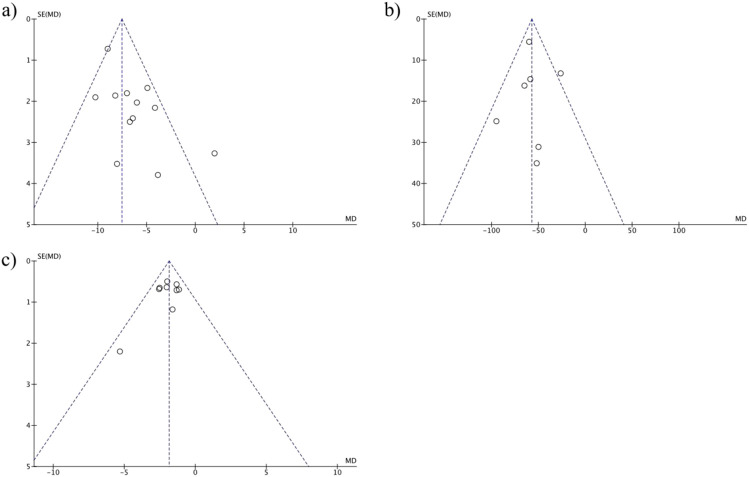
Funnel plots for physical characteristics: **(a)** Age, **(b)** Hypertension Duration, **(c)** BMI, assessing OR for postoperative hypertension.

### 3.7 Publication bias analysis

To assess potential bias from unreported studies, funnel plots and Egger’s regression tests were performed:

#### 3.7.1 Age

**FIGURE 7:**
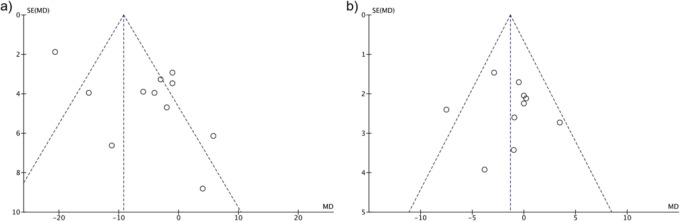
Funnel plots for Blood Pressure Markers: **(a)** SBP, **(b)** DBP, Estimating OR with 95% CI for postoperative hypertension.

The symmetric funnel plot (Figure 7) and Egger’s test (P = 0.27) indicated no significant publication bias, suggesting that smaller studies with non-significant results were not systematically omitted.

#### 3.7.2 BMI

**FIGURE 8:**
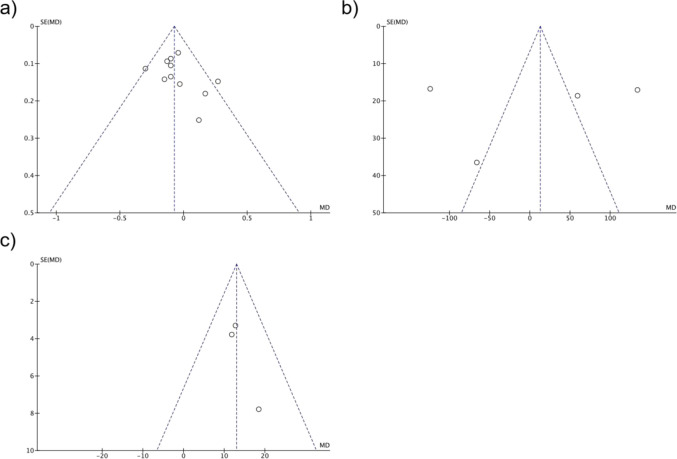
Funnel plots for biochemical markers: **(a)** Serum Potassium, **(b)** AAR, **(c)** eGFR, associating OR with postoperative hypertension risk.

Funnel plot symmetry (Figure 8) and Egger’s P = 0.38 confirmed no meaningful asymmetry, supporting the representativeness of included BMI data.

#### 3.7.3 Hypertension duration

The funnel plot (Figure 8) showed minimal asymmetry, with Egger’s test (P = 0.41) ruling out significant bias from unreported long-duration studies.

#### 3.7.4 SBP and ARR

Funnel plots (Figure 5) for SBP (Egger’s P = 0.19) and ARR (Egger’s P = 0.29) showed no significant deviations from symmetry, indicating that published results likely reflect the true effect distribution.

## 4 Discussion

This systematic review and meta-analysis identifies four robust risk factors for persistent hypertension after primary aldosteronism (PA) surgery: hypertension duration, body mass index (BMI), serum potassium, and estimated glomerular filtration rate (eGFR). These findings highlight the cumulative impact of aldosterone-mediated tissue damage on vascular and renal systems, (Armanini et al., 2020), providing a framework for preoperative risk stratification.

Prolonged hypertension duration reflects chronic aldosterone exposure, driving vascular remodeling (collagen deposition, endothelial dysfunction) and myocardial fibrosis via mineralocorticoid receptor activation (Bioletto et al., 2022; Picado et al., 2021). This aligns with prior studies showing that each additional year of hypertension increases postoperative persistence odds (Picado et al., 2021). The irreversible structural changes in chronic hypertension make blood pressure control difficult even after surgical adenoma removal (Cai et al., 2021; Wachtel and Fraker, 2021). Preoperative hypertension also signals underlying systemic inflammation and endothelial dysfunction, which sustain elevated blood pressure postoperatively (Huang-Fu et al., 2022; Pillai et al., 2020).

Higher BMI amplifies aldosterone’s hypertensive effects through mineralocorticoid receptor (MR)-mediated adipocyte dysfunction (Hall et al., 2021). Aldosterone promotes pro-inflammatory adipokine release (TNF-α, IL-6) and reduces adiponectin secretion, impairing insulin signaling and vascular health (Hall et al., 2014; Valensi, 2021; Shariq and McKenzie, 2020). This mechanism explains the strong association between obesity and postoperative hypertension, consistent with literature linking BMI to hypertension persistence (Gershuni et al., 2020; Chait and den Hartigh, 2020).

The meta-analysis found that higher preoperative serum potassium levels predict persistent hypertension post-surgery (Lee et al., 2021). This may seem counterintuitive, as classic PA is associated with hypokalemia, but advanced renal damage from chronic aldosterone excess (e.g., tubulointerstitial fibrosis) impairs renal potassium secretion, leading to normo- or hyperkalemia despite ongoing aldosterone action (Clase et al., 2020; Burnier and Damianaki, 2023). This paradox underscores that high potassium reflects irreversible renal injury—driven by prolonged aldosterone exposure—rather than mild aldosteronism. Such injury sustains hypertension via vascular remodeling and endothelial dysfunction (Bioletto et al., 2022; Cai et al., 2021). Clinically, preoperative potassium levels are essential for risk prediction: patients with hyperkalemia require tailored preoperative assessments and postoperative management, including pharmacological therapy and dietary interventions (Preda et al., 2020; Rodan and Huang, 2009; Palmer, 2020).

Lower preoperative eGFR, indicative of aldosterone-induced renal fibrosis, correlates with postoperative hypertension (Li et al., 2023). Even mild renal impairment signals irreversible damage that worsens BP control post-surgery (Kobayashi et al., 2020; Nakano et al., 2023; Chauhan et al., 2022). Mechanistically, renal impairment drives volume retention, electrolyte disturbances, and renin-angiotensin-aldosterone system dysregulation (Chauhan et al., 2022; Reincke et al., 2021), while limiting sodium-water excretion (De Bhailis and Kalra, 2022). Preoperative eGFR assessment is thus critical for identifying high-risk patients. Noting that only 3 of 12 studies reported eGFR changes, further research is needed to validate this association (Li et al., 2023).

Age, SBP, and AAR were not identified as risk factors due to high heterogeneity across studies (I^2^ >75% for AAR). DBP approached significance (P = 0.06), likely limited by small sample sizes and measurement heterogeneity (ambulatory vs. office BP) (De Bhailis and Kalra, 2022; Chen et al., 2021). The near-significant association aligns with biological plausibility, as DBP reflects aldosterone-induced small-vessel dysfunction (Chen et al., 2021).

All identified risk factors converge on aldosterone’s pathogenic pathways: prolonged exposure drives vascular/renal remodeling, obesity enhances aldosterone sensitivity, and elevated potassium/eGFR decline indicate advanced organ damage. This suggests that pre-existing tissue injury from chronic MR activation sustains hypertension post-surgery, even after adenoma removal (De Bhailis and Kalra, 2022; Chen et al., 2021).

Postoperative hypertension in primary aldosteronism is predicted by hypertension duration, BMI, serum potassium, and eGFR—markers of cumulative aldosterone exposure and renal injury. Clinically, these factors necessitate preoperative risk stratification to guide tailored management. Diastolic blood pressure trended toward significance (P = 0.06), hindered by small samples and measurement variability, warranting larger studies with standardized BP protocols (De Bhailis and Kalra, 2022; Chen et al., 2021). All risk factors converge on aldosterone-mediated mineralocorticoid receptor activation, driving vascular/renal remodeling and metabolic dysfunction. Future studies should investigate the role of MR antagonists in post-surgical PA patients, particularly those with high-risk features, to target residual aldosterone effects independent of blood pressure control (De Bhailis and Kalra, 2022; Chen et al., 2021).

This review has several limitations somehow: 1) Most included studies were observational, precluding causal inference; 2) Heterogeneity in BP measurement protocols (ABPM vs. office BP) and postoperative hypertension definitions; 3) Language restriction to English/Chinese, potentially introducing publication bias; 4) Incomplete adjustment for antihypertensive medications in some studies; 5) Limited data on long-term outcomes or gene-aldosterone interactions.

## 5 Conclusion

In this meta-analysis, we made investigations of the potential risks, covering different aspects of physical status, blood pressure, and some biochemical indicators. Through a set of strict inclusion criteria, the following risk factors were identified to be potentially correlate with postoperative hypertension: duration of hypertension, BMI, serum potassium levels, and eGFR. This meta-analysis provides the first comprehensive synthesis of risk factors for persistent hypertension after PA surgery, integrating data from 12 studies to resolve prior inconsistencies and establish an evidence-based framework for clinical risk stratification. However, the exact mechanisms still need further support from clinical practices and molecular evidence.

## Data Availability

The raw data supporting the conclusion of this article will be made available by the authors, without undue reservation.

## References

[B1] Armanini D. Sabbadin C. Andrisani A. Ambrosini G. Bordin L. (2020). Primary aldosteronism: involvement of sympathetic system in the persistence of hypertension after surgery. J. Clin. Hypertens. (Greenwich). 22 (9), 1616–1617. 10.1111/jch.13964 32810354 PMC8030013

[B2] BiLiGe W. Wang C. Bao J. Yu D. Min A. Hong Z. (2019). Predicting factors related with uncured hypertension after retroperitoneal laparoscopic adrenalectomy for unilateral primary aldosteronism. Medicine 98 (30), e16611. 10.1097/MD.0000000000016611 31348309 PMC6708826

[B3] Bioletto F. Bollati M. Lopez C. Arata S. Procopio M. Ponzetto F. (2022). Primary aldosteronism and resistant hypertension: a pathophysiological insight. Int. J. Mol. Sci. 23 (9), 4803. 10.3390/ijms23094803 35563192 PMC9100181

[B4] Burnier M. Damianaki A. (2023). Hypertension as cardiovascular risk factor in chronic kidney disease. Circulation Res. 132(8), 1050–1063. 10.1161/CIRCRESAHA.122.321762 37053276

[B5] Cai Z. Gong Z. Li Z. Li L. Kong W. (2021). Vascular extracellular matrix remodeling and hypertension. Antioxidants and Redox Signal. 34 (10), 765–783. 10.1089/ars.2020.8110 32460598

[B6] Chait A. den Hartigh L. J. (2020). Adipose tissue distribution, inflammation and its metabolic consequences, including diabetes and cardiovascular disease. Front. Cardiovasc. Med. 7, 22. 10.3389/fcvm.2020.00022 32158768 PMC7052117

[B7] Chauhan K. Schachna E. Libianto R. Ryan J. Hutton H. Fuller P. J. (2022). Screening for primary aldosteronism is underutilised in patients with chronic kidney disease. J. Nephrol. 35 (6), 1667–1677. 10.1007/s40620-022-01267-3 35195879 PMC9300536

[B8] Chen C. W. Tsai C. H. Hung C. S. Tsai I. J. Chiu Y. W. Chang C. C. (2021). Comparison of cystatin C-based and creatinine-based glomerular filtration rate in the prediction of postoperative residual hypertension in aldosterone-producing adenoma patients after adrenalectomy. Clin. Chim. Acta 520, 147–153. 10.1016/j.cca.2021.06.010 34116005

[B9] Chen Q. Sun X. Hu T. Wan J. Chen J. Chen Y. (2023). Construction and evaluation of nomogram model for predicting the risk of untreated hypertension in patients with primary hyperaldosteronism after surgery. Nursing Practice and Research 20(16), 2369–2376. 10.3969/j.issn.1672-9676.2023.16.001

[B10] Citton M. Viel G. Rossi G. P. Mantero F. Nitti D. Iacobone M. (2015). Outcome of surgical treatment of primary aldosteronism. Langenbecks Arch. Surg. 400 (3), 325–331. 10.1007/s00423-014-1269-4 25567077

[B11] Clase C. M. Carrero J.-J. Ellison D. H. Grams M. E. Hemmelgarn B. R. Jardine M. J. (2020). Potassium homeostasis and management of dyskalemia in kidney diseases: conclusions from a kidney disease: improving global outcomes (KDIGO) controversies conference. Kidney Int. 97 (1), 42–61. 10.1016/j.kint.2019.09.018 31706619

[B12] De Bhailis Á. M. Kalra P. A. (2022). Hypertension and the kidneys. Br. J. Hosp. Med. 83 (5), 1–11. 10.12968/hmed.2021.0440 35653320

[B13] Funder J. W. Carey R. M. Mantero F. Murad M. H. Reincke M. Shibata H. (2016). The management of primary aldosteronism: case detection, diagnosis, and treatment: an endocrine society clinical practice guideline. J. Clin. Endocrinol. Metab. 101 (5), 1889–1916. 10.1210/jc.2015-4061 26934393

[B14] Gershuni V. M. Herman D. S. Kelz R. R. Roses R. E. Cohen D. L. Trerotola S. O. (2020). Challenges in obesity and primary aldosteronism: diagnosis and treatment. Surgery 167 (1), 204–210. 10.1016/j.surg.2019.03.036 31542169

[B15] Hall J. E. Mouton A. J. da Silva A. A. Omoto A. C. M. Wang Z. Li X. (2021). Obesity, kidney dysfunction, and inflammation: interactions in hypertension. Cardiovasc. Res. 117 (8), 1859–1876. 10.1093/cvr/cvaa336 33258945 PMC8262632

[B16] Hall M. E. do Carmo J. M. da Silva A. A. Juncos L. A. Wang Z. Hall J. E. (2014). Obesity, hypertension, and chronic kidney disease. Int. J. Nephrol. Renov. Dis. 7, 75–88. 10.2147/ijnrd.S39739 PMC3933708 24600241

[B17] Huang-Fu Y. C. Du Y. Q. Yu L. P. Xu T. (2022). Risk factors of persistent hypertension in primary aldosteronism patients after surgery. Beijing Da Xue Xue Bao Yi Xue Ban. 54 (4), 686–691. 10.19723/j.issn.1671-167x.2022.04.017 35950393 PMC9385504

[B18] Kim R. M. Lee J. Soh E. Y. (2010). Predictors of resolution of hypertension after adrenalectomy in patients with aldosterone-producing adenoma. J. Korean Med. Sci. 25 (7), 1041–1044. 10.3346/jkms.2010.25.7.1041 20592896 PMC2890881

[B19] Kitamoto T. Omura M. Suematsu S. Saito J. Nishikawa T. (2018). KCNJ5 mutation as a predictor for resolution of hypertension after surgical treatment of aldosterone-producing adenoma. J. Hypertens. 36 (3), 619–627. 10.1097/hjh.0000000000001578 29016532

[B20] Kobayashi Y. Haze T. Yano Y. Tamura K. Kurihara I. Ichijo T. (2020). Associations between changes in plasma renin activity and aldosterone concentrations and changes in kidney function after treatment for primary aldosteronism. Kidney Int. Rep. 5 (8), 1291–1297. 10.1016/j.ekir.2020.06.012 32775828 PMC7403537

[B21] Lee M.-J. Sun C.-Y. Lu C.-C. Chang Y. S. Pan H. C. Lin Y. H. (2021). Urinary sodium potassium ratio is associated with clinical success after adrenalectomy in patients with unilateral primary aldosteronism. Ther. Adv. Chronic Dis. 12, 2040622321990274. 10.1177/2040622321990274 33633824 PMC7887682

[B22] Li Z. He Y. Zhang Y. Chen G. Zheng Y. Guo Y. (2023). Predictive model for persistent hypertension after surgical intervention of primary aldosteronism. Sci. Rep. 13 (1), 11868. 10.1038/s41598-023-39028-2 37481689 PMC10363150

[B23] Moher D. Liberati A. Tetzlaff J. Altman D. G. PRISMA Group (2009). Preferred reporting items for systematic reviews and meta-analyses: the PRISMA statement. Ann. Intern. Med. 151 (4), 264–W64. 10.7326/0003-4819-151-4-200908180-00135 19622511

[B24] Mourad J. J. Girerd X. Milliez P. Lopez-Sublet M. Lejeune S. Safar M. E. (2008). Urinary aldosterone-to-active-renin ratio: a useful tool for predicting resolution of hypertension after adrenalectomy in patients with aldosterone-producing adenomas. Am. J. Hypertens. 21 (7), 742–747. 10.1038/ajh.2008.175 18443567

[B25] Nakano Y. Murakami M. Hara K. Fukuda T. Horino M. Takeuchi A. (2023). Long‐term effects of primary aldosteronism treatment on patients with primary aldosteronism and chronic kidney disease. Clin. Endocrinol. 98 (3), 323–331. 10.1111/cen.14849 36367014

[B26] Palmer B. F. (2020). Potassium binders for hyperkalemia in chronic kidney disease—diet, renin-angiotensin-aldosterone system inhibitor therapy, and hemodialysis. *Mayo Clin. Proc*. 2020/02/01/ 95 (2), 339–354. 10.1016/j.mayocp.2019.05.019 31668450

[B27] Picado O. Whitfield B. W. Khan Z. F. Jeraq M. Farrá J. C. Lew J. I. (2021). Long-term outcome success after operative treatment for primary aldosteronism. Surgery 169 (3), 528–532. 10.1016/j.surg.2020.07.046 32948336

[B28] Pillai P. R. Griffith M. Schwarcz M. D. Weiss I. A. (2020). Primary aldosteronism: cardiovascular risk, diagnosis, and management. Cardiol. Rev. 28 (2), 84–91. 10.1097/CRD.0000000000000281 31868768

[B29] Preda C. Teodoriu L. C. Placinta S. Grigorovici A. Bilha S. Ungureanu C. M. (2020). Persistent severe hyperkalemia following surgical treatment of aldosterone-producing adenoma. J. Res. Med. Sci. 25 (1), 17. 10.4103/jrms.JRMS_603_19 32174989 PMC7053163

[B30] Reincke M. Bancos I. Mulatero P. Scholl U. I. Stowasser M. Williams T. A. (2021). Diagnosis and treatment of primary aldosteronism. lancet Diabetes and Endocrinol. 9 (12), 876–892. 10.1016/S2213-8587(21)00210-2 34798068

[B31] Rodan A. R. Huang C.-L. (2009). Distal potassium handling based on flow modulation of maxi-K channel activity. Curr. Opin. Nephrol. Hypertens. 18 (4), 350–355. 10.1097/MNH.0b013e32832c75d8 19448535 PMC3151167

[B32] Shariq O. A. McKenzie T. J. (2020). Obesity-related hypertension: a review of pathophysiology, management, and the role of metabolic surgery. Gland. Surg. 9 (1), 80–93. 10.21037/gs.2019.12.03 32206601 PMC7082272

[B33] Utsumi T. Kamiya N. Endo T. Yano M. Kamijima S. Kawamura K. (2014). Development of a novel nomogram to predict hypertension cure after laparoscopic adrenalectomy in patients with primary aldosteronism. World J. Surg. 38 (10), 2640–2644. 10.1007/s00268-014-2612-1 24831672

[B34] Valensi P. (2021). Autonomic nervous system activity changes in patients with hypertension and overweight: role and therapeutic implications. Cardiovasc. Diabetol. 20 (1), 170. 10.1186/s12933-021-01356-w 34412646 PMC8375121

[B35] Wachtel H. Fraker D. L. (2021). Therapeutic outcomes with surgical and medical management of primary aldosteronism. Curr. Cardiol. Rep. 23 (7), 89. 10.1007/s11886-021-01516-0 34081226 PMC8341292

[B36] Worth P. J. Kunio N. R. Siegfried I. Sheppard B. C. Gilbert E. W. (2015). Characteristics predicting clinical improvement and cure following laparoscopic adrenalectomy for primary aldosteronism in a large cohort. Am. J. Surg. 210 (4), 702–709. 10.1016/j.amjsurg.2015.05.033 26323999

[B37] Wu J. (2023). The related factors of postoperative blood pressure in primary aldosteronism. Master’s. 10.27108/d.cnki.ghelu.2023.001051

[B38] Wu J. Tang Z. Zhang W. Ling D. Hou R. Li X. (2006). Logistic regression analysis of factors associated with hypertension after operation of aldosterone producing adnoma. Chin. J. Pract. Intern. Med. (22), 1802–1804.

[B39] Wu V.-C. Chueh S.-C. Chang H.-W. Lin L.-Y. Liu K.-L. Lin Y.-H. (2009). Association of kidney function with residual hypertension after treatment of aldosterone-producing adenoma. Am. J. Kidney Dis. 54 (4), 665–673. 10.1053/j.ajkd.2009.06.014 19628318

[B40] Zhou Y. Zhang M. Ke S. Liu L. (2017). Hypertension outcomes of adrenalectomy in patients with primary aldosteronism: a systematic review and meta-analysis. BMC Endocr. Disord. 17 (1), 61. 10.1186/s12902-017-0209-z 28974210 PMC5627399

